# Efficacy and Safety of Diet Therapies in Children With Autism Spectrum Disorder: A Systematic Literature Review and Meta-Analysis

**DOI:** 10.3389/fneur.2022.844117

**Published:** 2022-03-14

**Authors:** Yuping Yu, Jinyue Huang, Xiaofang Chen, Jia Fu, Xinhui Wang, Linjie Pu, Chunyu Gu, Chunquan Cai

**Affiliations:** ^1^Graduate College of Tianjin Medical University, Tianjin, China; ^2^Tianjin Children's Hospital (Children's Hospital of Tianjin University), Tianjin, China; ^3^Tianjin Pediatric Research Institute, Tianjin, China; ^4^Tianjin Key Laboratory of Birth Defects for Prevention and Treatment, Tianjin, China; ^5^Department of Neurosurgery, Tianjin Children's Hospital, Tianjin, China

**Keywords:** Autism Spectrum Disorders, gluten-free and casein-free diet, gluten-free diet, ketogenic diet, meta-analysis, childhood

## Abstract

**Objective:**

Autism Spectrum Disorder is a neurodevelopmental disorder, with a rapid increase in recognition over the past decade. Interest in alternative therapies is growing annually, such as dietary therapies including gluten-free and/or casein-free diet, and the ketogenic diet. However, there is no consensus on the efficacy and safety of dietary therapy in children with ASD up to now. This study aimed to assess the efficacy and safety of these diet interventions for children with ASD based on a meta-analysis of global data.

**Methods:**

Seven databases (Cochrane Library, PubMed, EMBASE, Web of Science, VIP, CNKI, and Wanfang) were searched according to the established inclusion criteria, from the inception of the databases to August 18, 2021. The Cochrane Bias risk assessment tool was intended to assess the quality of the included studies. Review Manager 5.4 software was used as an efficacy analysis tool of the included studies, taking the core autistic symptoms and scales of ASD as therapeutic efficacy evaluations.

**Results:**

In total, 7 RCTs with 338 participants were finally obtained. All studies assessed the association between core autistic symptoms and therapeutic diet, showing a statistically significant effect (standard mean difference (SMD) of −0.51, 95% confidence interval (Cl): −0.81 to −0.21), in which two studies which followed the GFD diet reported significant reductions in social behaviors (SMD of−0.41, 95% Cl: −0.75 to −0.06), showing no correlation with the length of the interventions (*P* < 0.05). Two studies were performed in KD diet suggested a significant effect in core symptoms (SMD of −0.67, 95% Cl: −1.04 to −0.31). No statistically significant changes were observed in the GFCF diet, GFD diet, cognition, communication, and stereotypical behaviors subgroups (all *P* > 0.05).

**Conclusion:**

The results of a meta-analysis suggest that diet therapies can significantly ameliorate core symptoms of ASD, and GFD diets are conducive to improving social behaviors. Although the results suggest the effectiveness of dietary therapy for ASD, limited by the small sample size of RCTs, more well-designed, and high-quality clinical trials are needed to validate the above conclusions.

**Systematic Review Registration:**

https://www.crd.york.ac.uk/PROSPERO/, identifier: CRD42021277565.

## Introduction

Autism Spectrum Disorder (ASD) is a neurodevelopmental disorder, which affects several spheres of normal mental development with an onset in the first few years of life, usually prior to age 3 (1–3). Children with ASD are characterized by deficits in social interaction and social communication, together with the presence of repetitive, restricted patterns of behavior, interests, or activities present during early periods of development (4). Otherwise, gastrointestinal (GI) disorders are a common comorbidity in patients with ASD. American Centers for Disease Control and Prevention (CDC) estimates that about 1 in 54 children has been identified with ASD (or 18.5 per 1,000 8-year-olds), with a male-to-female ratio of 4-to-1 (5). It is challenging and costly work to care for children with ASD, with an estimated lifetime cost per affected child of $2.4 million in the US(6). Hence, the growing incidence of people classed on the ASD brings a great challenge to families, schools, medical systems, and society.

As for ASD treatment for children and adolescents, the education and behavioral services therapies are primarily treated, and medication is the important adjunct (7). Behavioral interventions include high-intensity applied behavior analysis (ABA), early intensive behavioral intervention (EIBI), and social skills interventions (3). Currently, there are no drugs approved for the treatment of core symptoms of ASD, and it has been reported that children with ASD are generally more susceptible to side effects of psychoactive medications than their age-matched, neurodevelopmentally normal peers (8). Despite this, psychopharmacological interventions are undertaken by nearly half of diagnosed children with ASD, most commonly with stimulants, alpha-2 agonists, antipsychotics, anticonvulsants, and antidepressants (9). Therefore, alternative therapies are needed. Dietary interventions, including gluten-free and casein-free diet (GFCF), gluten-free diet (GFD), and ketogenic diet (KD), come into view as an alternative therapy for ASD. It is generally believed that a specific diet can help to alleviate gastrointestinal and behavioral symptoms for children with ASD.

The GFD is the dietary exclusion of gluten or gluten content of <20 mg/kg, including natural gluten-free foods (legumes, fruits and vegetables, unprocessed meats, fish, eggs, and dairy products) and alternatives to gluten-based cereals. Casein-free diets involve the dietary exclusion of casein, a protein found in dairy and other foods containing dairy or lactose products. GFCF is a dietary protocol combining GFD and casein-free diets. KD is a formula diet rich in fat, moderate in protein, and low in carbohydrates, with fat as the main source of calories. In recent years, these diets as a non-traditional treatment approach have been used for patients with ASD. It is currently believed that opioid peptides produced by the gastrointestinal tract upon digestion of gluten and casein can pass through the mucosa and cross the blood-brain barrier to reach the central nervous system, and then affect brain function as well as contribute to the development of ASD (10, 11). So far, the mechanism by which KD treatment improves ASD symptoms is uncertain. One study demonstrates that KD consumption triggers gut microbiota remodeling in a BTBR mouse model of ASD, which may be a potential ASD therapeutic mechanism of KD (12). Another study claims that that the BTBR mouse model of ASD exhibits increased cortical excitability and that KD dietary therapy can reverse this abnormality (13). Nonetheless, the effectiveness and safety of those nutritional interventions for ASD are uncertain, and no consensus exists regarding optimal nutritional therapy (14–16). The evidence about the use of the GFCF/GD/KD diet is discordant, some studies showed no significant impact of the diets for children with ASD (17–20), on the contrary, other studies indicated a beneficial influence (21–23). In conclusion, evidence about these diets and autism is scarce, and more studies are necessary to evaluate and support the effectiveness and safety. So far, several studies evaluated the effects of a GFCF diet on individuals with ASD (24, 25) with systematic and quantitative analysis. Keller et al. (24) identified six relevant RCTs, and the result showed no effect of a GFCF diet on clinician-reported autism core symptoms, parent-reported functional level, or behavioral difficulties. On the contrary, Quan et al. (25) indicated that a GFCF diet can reduce stereotypical behaviors and improve the cognition of children with ASD within a total of eight studies. Hence, this meta-analysis aimed to reevaluate the efficacy and safety of a GFCF diet, and evaluate the effectiveness of a KD diet for individuals with ASD.

## Methods

This systematic review and meta-analysis was carried out corresponding to the Cochrane Collaboration recommendations (26) and performed according to the GRADE (Grades of Recommendation, Assessment, Development, and Evaluation approach) (27), as well as adheres to the PRISMA (Preferred Reporting Items for Systematic Reviews and Meta-Analyses) guidelines (28, 29) (PRISMA checklist is provided in the Supplementary Table S1). Moreover, The PICOS (Participants, Intervention/exposure, Comparison, Outcomes, Study design) criteria were used to structure this systematic (30). The protocol of the study was registered on PROSPERO (CRD42021277565).

### Literature Search Strategy

Two independent coworkers searched four English databases (Cochrane Library, Web of Science, PubMed, and EMBASE) and three Chinese databases (VIP, Wanfang, and CNKI for randomized controlled trials (RCTs) published from the inception of the databases to August 18, 2021. The search strategies were (“Child Development Disorders, Pervasive” OR “Autism Spectrum Disorder” OR “ASD” OR “PDD” OR “Autism” OR “Autistic Disorder” OR “Autism Spectrum Disorder” OR “Asperger Syndrome” OR “Asperger Disorder” OR “Autistic Syndrome”) AND (“therapeutic diet” OR “ketogenic diet” OR “gluten-free diet” OR “gluten/casein-free diet” OR “Ketogenic Diets” OR “Diets, Ketogenic”) AND (“randomized controlled trial” OR “controlled clinical trial” OR “randomized” OR “placebo” OR “randomly” OR “trial” OR “groups” OR “RCT”). No restriction was taken on the language, but studies carried out on adults and animals were excluded. Reference tables of all meta-analyses, involved reviews, and obtained papers were manually searched to check for studies that had not appeared previously. Inconsistency was resolved by consensus.

### Inclusion and Exclusion Criteria

Inclusion criteria in the meta-analysis were the following: (1) the diagnosis of ASD according to the International Classification of Diseases (ICD) or the diagnostic criteria of the Diagnostic Statistical Manual (DSM); (2) Age range was 2 18 years (from the earliest age of a valid diagnosis until they come of age); (3) the children in the intervention group received therapeutic diets (including KD, GFD, and GFCF); (4) the patients in the control group were treated with regular diet or balanced nutrition diet; (5) the clinical follow-up was required to be at least 6 weeks; (6) the therapeutic efficacy was assessed by Core autism symptoms reported by clinician or observer; (7) RCTs.

Exclusion criteria in our research were as follows: (1) Not RCTs, including abstracts only, reviews, conference proceedings, case reports, animal studies, and non-clinical studies; (2) No available date; (3) Duplicated studies or repeated analysis.

### Study Selection and Data Extraction

According to the inclusion and exclusion criteria, two authors screened titles and abstracts independently to identify potential articles. Another two authors selected and decided the final studies included in the analysis by screening the full texts of the potential articles.

Two researchers evaluated study details from all included studies and extracted data using standardized forms. The data included the following items: first author, year of publication, country, sample size, age, gender, interventions, study design, diagnostic criteria utilized, exclusion criteria of patients, follow-up, outcomes, mean and standard deviation, and side effects. In crossover trials the latter period after crossover was excluded, to avoid the potential presence of carryover effects of treatment from the first period (26).

Disagreements arising during this process were resolved and reached consensus by collective discussion.

### Quality Assessment and Risk of Bias

The quality and risk of bias critical appraisals of included studies were assessed independently with conflicts resolved through discussion by three researchers on the basis of the Cochrane risk of bias tool for RCTs (31). The Cochrane Collaboration's tool provides seven quality domains: random sequence generation, allocation concealment, blinding of participants and personnel, blinding of outcome assessment, incomplete outcome data, selective reporting, and other bias. Funnel plots were used to investigate publication bias.

### Data Analyses

In our research, we focused on the overall core autism symptoms of ASD and the four main core symptom subgroups including communication difficulties, social disorders, stereotypical behaviors, and cognition. In addition, we conducted subgroup analyses according to different intervention durations (ranging from 6 weeks to 12 months) and interventions (GFCF, GFD, and KD).

Review Manager 5.4 software (26) was used for the meta-analysis. Because all outcomes of included studies were continuous variables and the scales for each article were different measurements, the effect size was assessed as the standardized mean difference (SMD; 95% CI), while *P* < 0.05 suggested a significant difference. The heterogeneity test was undertaken by the Q test and I^2^ test. If a *P*-value > 0.1 (*Q* test) as well as I^2^ <50% (I^2^ test), heterogeneity was considered to be meaningless. Random effects model was used in this study. Sensitivity analysis was used to evaluate the stability and reliability of the combined results of each meta-analyses in this study. Sensitivity analysis was performed by removing one study at a time. Funnel plots were plotted to visually detect publication bias.

## Results

### Search Results and Study Characteristics

**Figure 1:**
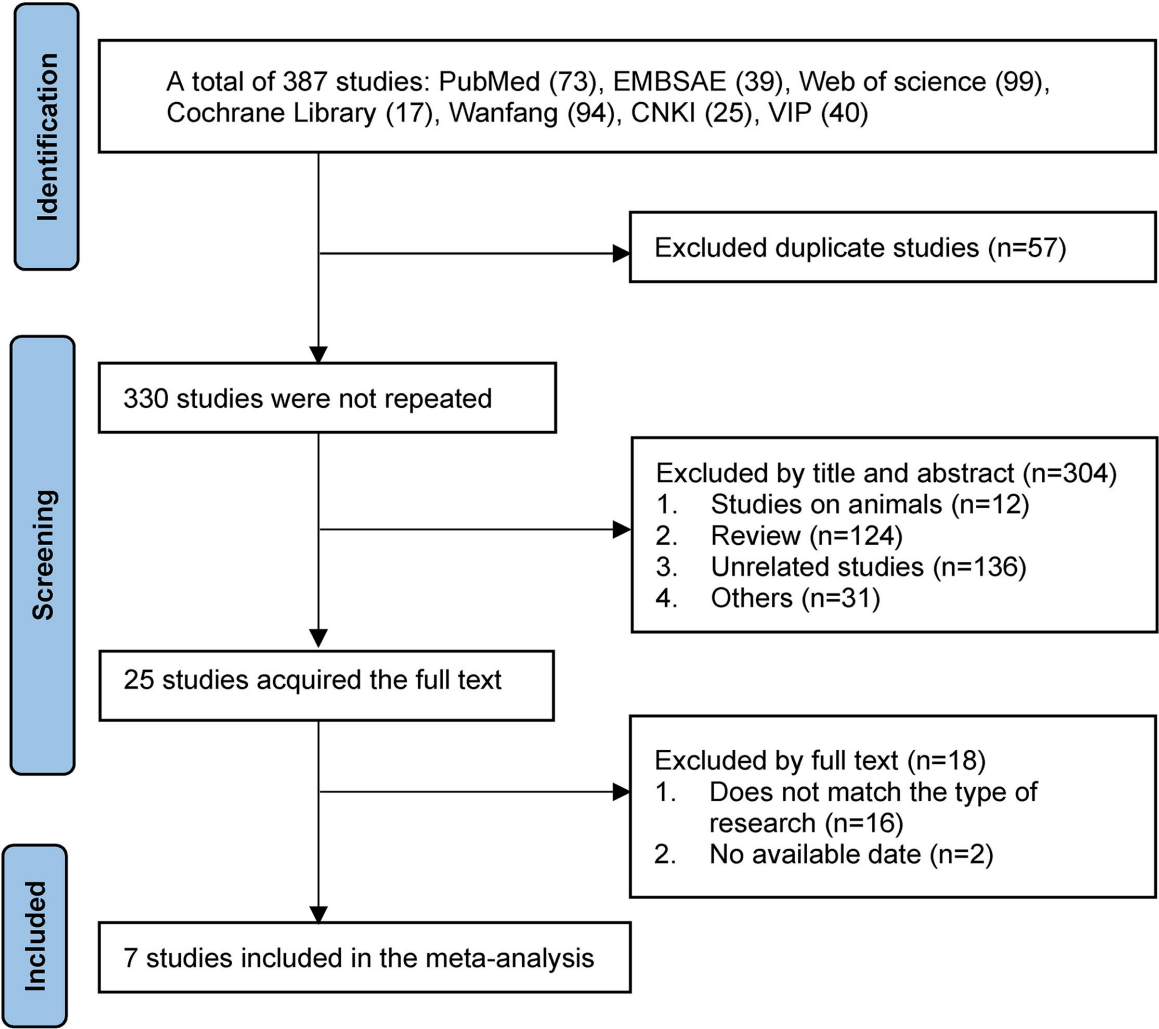
Flow diagram of selected studies. CNKI, China National Knowledge Infrastructure; VIP, China Science and Technology Journal Database.

A total of 387 articles were obtained by searching seven databases, including 228 in English and 159 in Chinese. Fifty seven duplicates identified by Endnote software were excluded, 304 studies were excluded by screening the titles and abstracts, and the remaining potential 25 studies were searched at a full-text level. Finally, six English articles and one Chinese article with a total of 7 RCTs were included in the meta-analysis (17–19, 21–23, 32). In this regard, studies of Navarro et al. (33) and Whiteley et al. (34) were excluded due to the lack of necessary data in the articles, without contacting the authors, though they met the main inclusion criteria. The flow diagram of selected studies was summarized in Figure 1.

Our analysis included seven studies, all of them were RCTs, in which two were crossover RCTs [Elder et al. (17) and González-Domenech et al. (32)]. Them were performed in the United States (17), Egypt (21), Iran (18), Spain (32), Norway (22), Poland (19), and China (23), respectively. Sample size ranged from 7 to 50, age differed from 2 to 18 years, and duration varied from 6 weeks to 10 months. In total, this research pooled results from 338 patients with ASD, including 171 intervention subjects and 167 age-sex-matched controls. Most patients were diagnosed with ASD by DSM-4 (17), DSM-5 (19, 21, 23), ICD-10 (19, 32), and Autism diagnostic interview-Revised (ADI-R) (18), while Knivsberg et al. (22) did not describe the methods of patients' diagnosis in detail. Three studies investigated the effectiveness of a GFCF diet in individuals with ASD (17, 22, 32), two studies assessed the efficacy of a GFD for children with ASD (18, 19), one study surveyed the influence of a KD on patients with ASD (23), while the other one publication examined the effects of KD vs. GFCF in children with ASD at the same time(21). Besides the study reported by Knivsberg et al. the remaining six studies described the excluded diet conditions of intervention groups, including dietary intolerances, nutritional or metabolic diseases, abnormal growth, and development, or other major medical problems. The further characteristics of included studies were summarized in Table 1.

**Table 1:** The characteristics of the included studies in the meta-analysis.

**References**	**Region**,	**Sample size**	**Sample size**	**Male**	**Mean age (years)**,	**Mean age (years)**,	**Intervention**	**Control**	**Duration**
	**Country**	**intervention group**	**control group**	**(%)**	**intervention group**	**control group**			
Smith et al. (13)	USA	7	7	80.0	7.32	7.32	GFCF	Regular diet	6 weeks
Elder et al. (17)	Egypt	25	15	73.3	5.29	5.29	MAD, GFCF	Balanced nutrition	6 months
Karhu et al. (14)	Iran	38	38	73.6	7.84	8.00	GFD	Regular diet	6 weeks
Page et al. (28)	Spain	15	16	54.1	8.80	9.10	GFCF	Regular diet	6 months
Ghalichi et al. (18)	Norway	10	10	NA	7.60	7.20	GFCF	Regular diet	12 months
Piwowarczyk et al. (19)	China	50	50	77.0	3.59	3.61	KD	Regular diet	10 weeks
Baspinar et al. (15)	Poland	26	31	84.8	3.75	3.83	GFD	GD	6 months
**References**	**Study Design**		**Inclusion Criteria**		**Exclusion Criteria**			**Main assessment Tools**	
Smith et al. (13)	Double-blinded, crossover RCT		Diagnosis of ASD according to DSM-4		Patients with medical histories and/or physical examinations indicated that they had physical or sensory impairments or significant medical problems, including celiac disease			CARS score	
Elder et al. (17)	RCT		Diagnosis of ASD according to DSM-5		Patients with fat metabolism disorders, dyslipidemia, pyruvate carboxylase deficiency, porphyria, presence of kidney stones, liver disease, feeding problems or failure to thrive, gastroesophageal reflux, poor oral intake, cardiomyopathy, or chronic metabolic acidosis			CARS score	
Karhu et al. (14)	RCT		Diagnosis of ASD according to ADI-R by a psychologist		Patients not diagnosed as ASD according to ADI-R by a psychologist, feeding difficulties based on parent report, or inpatients and children with additional illnesses or abnormalities			CARS-2 score	
Page et al. (28)	Crossover RCT		Diagnosis of ASD according to ICD-10		Patients diagnosed with an allergy to gluten or casein; patients who had previously excluded gluten and/or casein from their diet; patients who were likely to not adhere to the diet properly			ATEC scale	
Ghalichi et al. (18)	RCT		Diagnosis of both ASD and abnormal urinary peptide patterns		NA			DIPAB autistic traits	
Piwowarczyk et al. (19)	RCT		Diagnosis of ASD according to DSM-5		Patients with nutritional and metabolic diseases, abnormal growth and development, or other significant medical problems.			ABC score	
Baspinar et al. (15)	Single-Blinded, RCT		Diagnosis of ASD according to DSM-5 or ICD-10		Patients with celiac disease, wheat allergy, inability to Cooperate, malnutrition, or presence of disease(s) influencing behavior, feeding, or growth			ADOS-2	

*GFCF, gluten-free and casein-free diet; MAD, modified ketogenic diet; KD, ketogenic diet; GFD, gluten-free diet; GD, gluten-containing diet. ASD, Autism spectrum disorder; DSM-4/5, the Diagnostic and Statistical Manual of Mental Disorders, Fourth/Fifth Edition; ADI-R, Autism diagnostic interview-Revised; ICD-10, the tenth edition of the International Classification of Diseases; GARS, Gilliam Autism Rating Scale; ATEC, Autism Treatment Evaluation Checklist; DIPAB, a standardized Danish scheme to evaluation behavior; ABC, Autism children behavior checklist; ADOS-2, Autism Diagnostic Observation Schedule, Second Edition; NA, not available*.

### Quality Assessment and Risk of Bias

**Figure 2:**
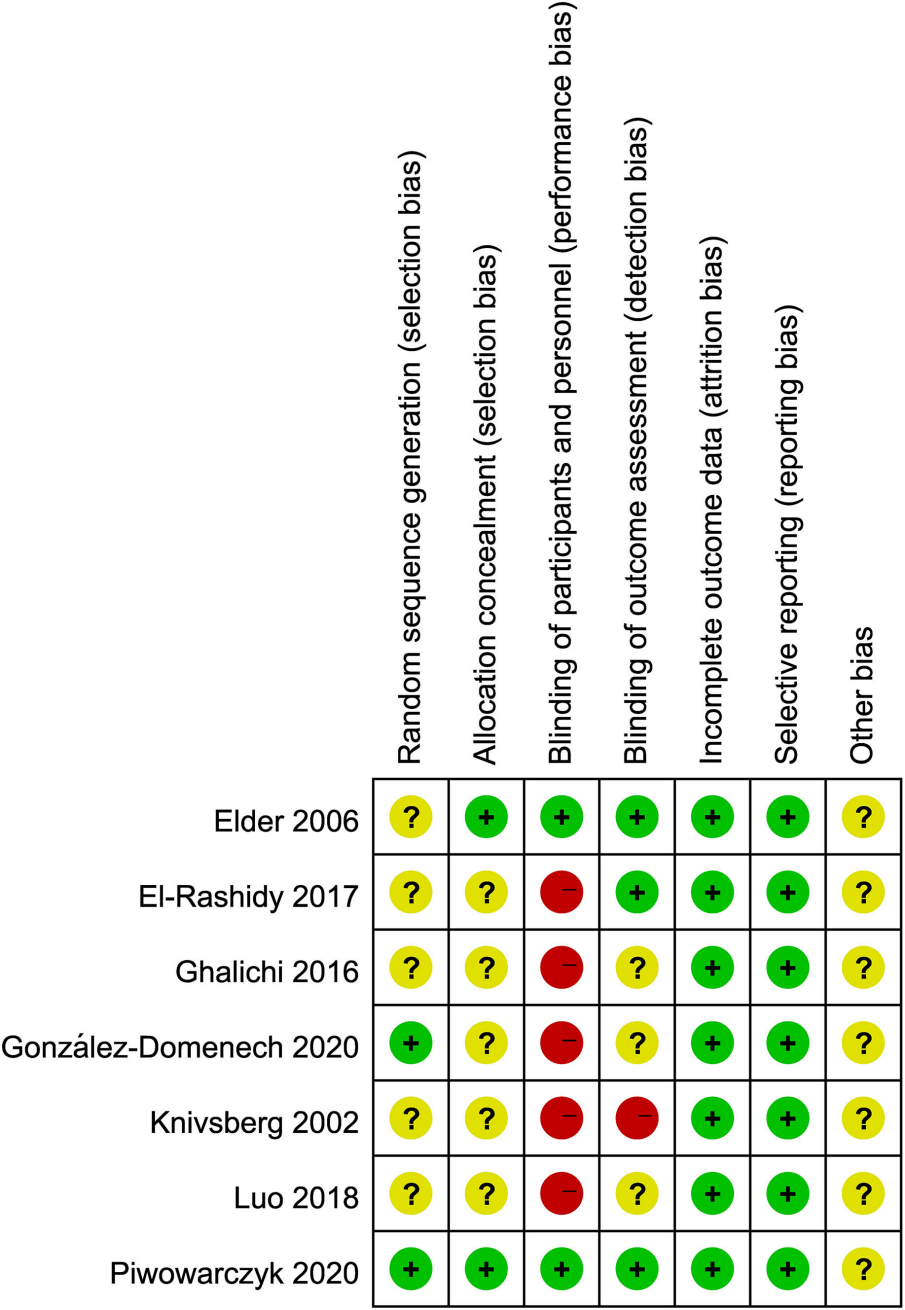
Risk of bias summary.

The quality assessment of the studies included in the meta-analysis was shown in Figure 2. A total of 7 RCTs were evaluated for quality analysis. The highest risk of bias was in the blinding (or lack thereof) of participants and personnel (performance bias), as well as blinding of outcome assessment (detection). Only in two studies (17, 19) were the method of random sequence generation for details described, and in the other studies, the process of random allocation was not described. It was not clear whether there were other biases among the included studies. A visual inspection of the evidence base by means of a funnel plot (Supplementary Figure S1) did not show any clear publication bias.

### Sensitivity Analysis

A sensitivity analysis was performed to assess the results of our research for clinician-reported core symptoms, duration, different interventions, stereotypical behaviors, social behaviors, cognition, and communication using Review Manager 5.4 software. The effect of each study on the pooled results was evaluated by excluding a single study sequentially. Most results of sensitivity analysis showed stability effect, while the study performed by Elder et al. (17) may have influenced the GFCF diets results of the meta-analysis.

### Results of the Meta-Analysis

**Figure 3:**
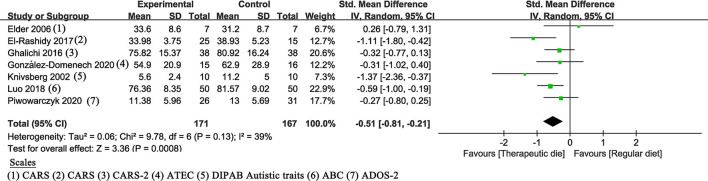
Meta-analysis results and scales for clinician-reported core symptoms. GARS (-2), Gilliam Autism Rating Scale (Second Edition); ATEC, Autism Treatment Evaluation Checklist; DIPAB, a standardized Danish scheme to evaluate behavior; ABC, Autism children behavior checklist; ADOS-2, Autism Diagnostic Observation Schedule, Second Edition.

All included studies assessed the association between therapeutic diet and clinician-reported core symptoms of ASD and showed a statistically significant effect (SMD of −0.51, 95% Cl: −0.81 to −0.21, *P* = 0.0008) (Figure 3). The heterogeneity (*P* = 0.13 and *I*^2^ =39%) was considered to be meaningless.

### Subgroup Analysis

#### Dietary Intervention

**Figure 4:**
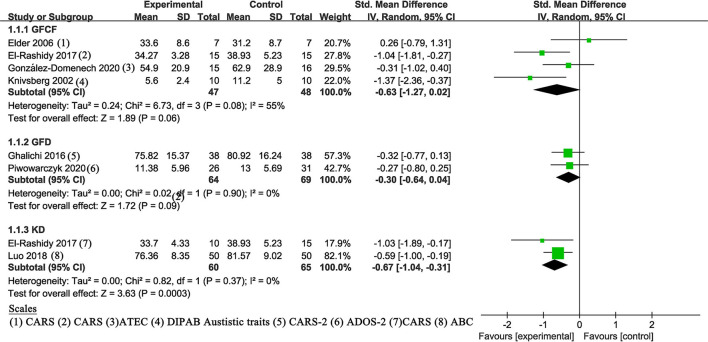
Meta-analysis results and scales for dietary intervention. GFCF, gluten-free and casein-free diet; GFD, gluten-free diet; KD, ketogenic diet; GARS (-2), Gilliam Autism Rating Scale (Second Edition); ATEC, Autism Treatment Evaluation Checklist; DIPAB, a standardized Danish scheme to evaluation behavior; ADOS-2, Autism Diagnostic Observation Schedule, Second Edition; ABC, Autism children behavior checklist.

A fixed-effects meta-analysis of different dietary interventions (GFCF, GFD, and KD) was performed (Figure 4). The results of KD (21, 23) (SMD of −0.67, 95% Cl: −1.04 to −0.31, *P* = 0.0003) indicated statistically significant improvement in clinician-reported core symptoms. No statistically significant changes were observed in GFCF (*P* = 0.06) and GFD (*P* = 0.09). Between-study heterogeneity was null in GFD and KD subgroups (both *I*^2^ = 0%), while the GFCF subgroup had moderate heterogeneity (*P* = 0.08, *I*^2^ = 55%).

#### Duration

**Figure 5:**
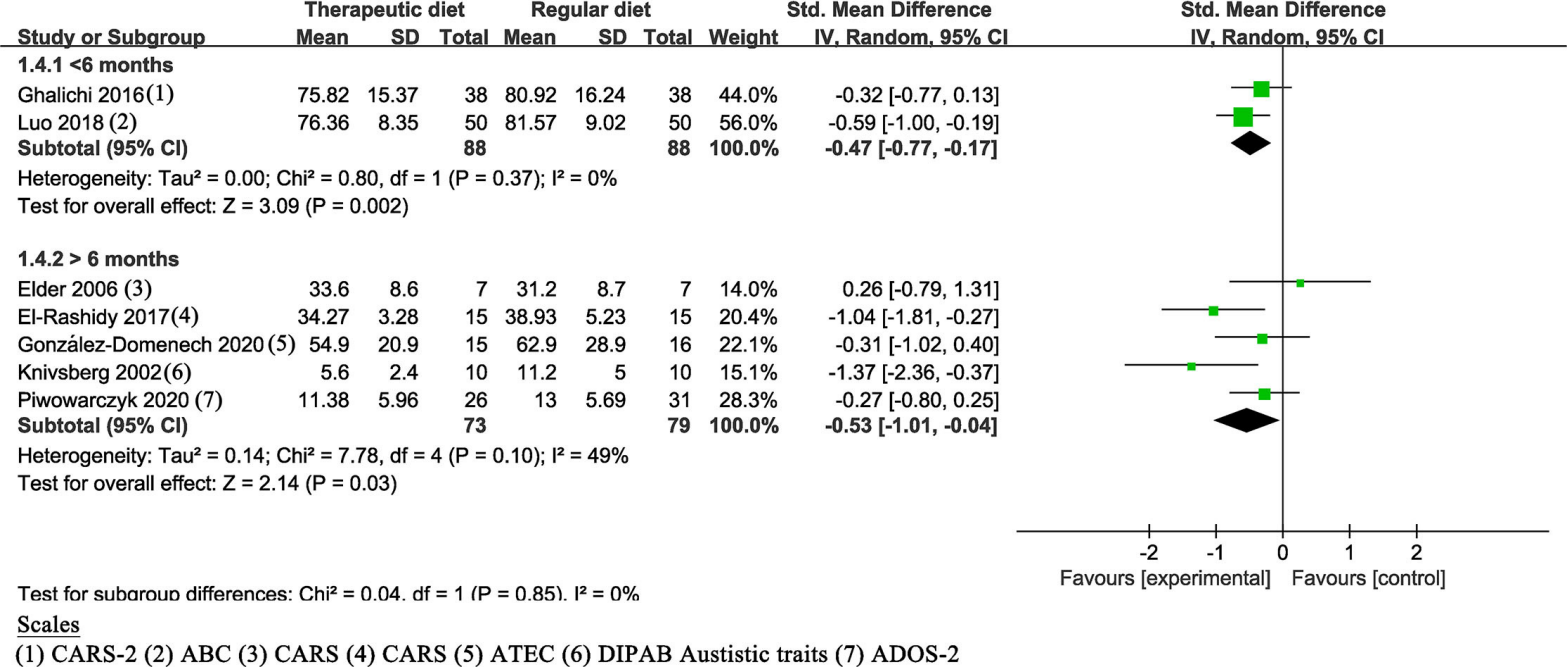
Meta-analysis results and scales for the duration. GARS (-2), Gilliam Autism Rating Scale (Second Edition); ABC, Autism children behavior checklist; ATEC, Autism Treatment Evaluation Checklist; DIPAB, a standardized Danish scheme to evaluate behavior; ADOS-2, Autism Diagnostic Observation Schedule, Second Edition.

In the meta-analysis, the intervention duration varied from 6 weeks to 10 months. The duration of five studies (17, 19, 21, 22, 32) was ≥6 months, whereas the other two studies (18, 23) were <6 months. All results showed a statistically significant effect: ≥6 months (SMD of −0.53, 95% Cl: −1.01 to −0.04, *P* = 0.03); <6 months (SMD of −0.47, 95% Cl: −0.77 to −0.17, *P* = 0.002) (Figure 5).

#### Social Behaviors

**Figure 6:**
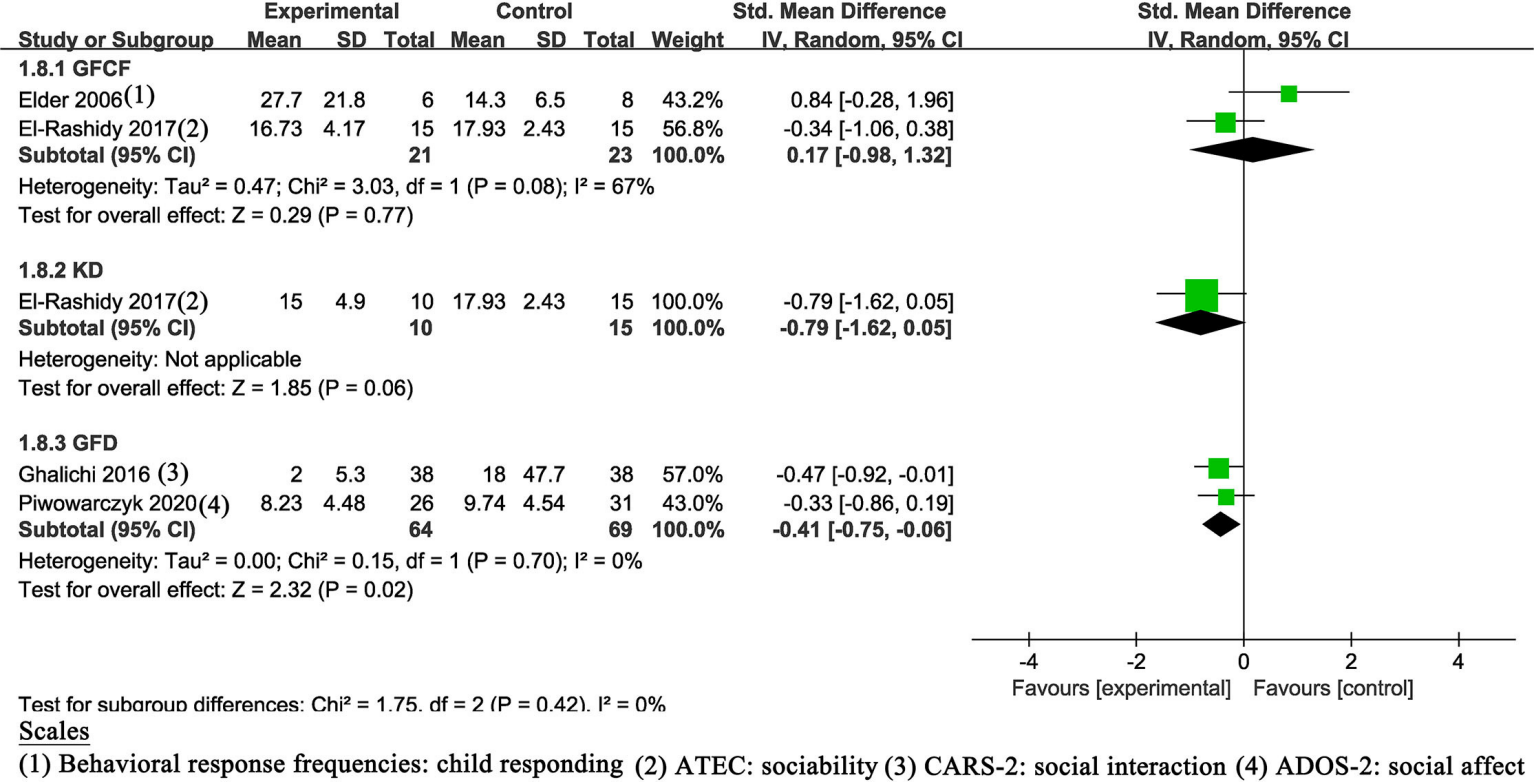
Meta-analysis results and scales for social behaviors. ATEC, Autism Treatment Evaluation Checklist; GARS-2, Gilliam Autism Rating Scale, Second Edition; ADOS-2, Autism Diagnostic Observation Schedule, Second Edition.

Four trials (17–19, 21) described details of social behaviors, two of which followed the GFCF diet, two followed the GFD diet, and one of which was treated with the KD diet. The result of the GFD diet (SMD of −0.41, 95% Cl: −0.65 to −0.06, *P* = 0.02) indicated statistically significant improvement (Figure 6). The other two results showed no statistically significant improvement (*P* > 0.05).

#### Others

Three studies (19, 21, 22) investigated cognition, five trials (17–19, 21, 22) described communication, and five studies assessed stereotypical behaviors (17–19, 21, 22). However, there were no statistically significant changes were observed (all *P* > 0.05) (Supplementary Figures S2–S4).

Only four trials reported side effects such as GI discomfort and other changes. Since only one study used the scale (the ROME 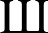 questionnaire, a valuable tool for assessment of GI symptoms) for GI system evaluation, no meta-analysis was conducted. Ghalichi et al. (18) assessed GI outcomes according to the ROME 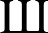 questionnaire, the observed GFD group had significant improvement in stomachache, bloating, and constipation, whereas there were no significant differences in the RD group. González-Domenech et al. (32) reported there were no significant differences in the nutritional variables (calcium, vitamin D, ferritin, folic acid, IGF-1, and hematocrit), weight and height, history of GI, and eating disorders between groups. Luo et al. (23) declared there were no adverse reactions observed due to a special dietary cycle (4 weeks KD + 2 weeks regular diet + 4 weeks KD). Piwowarczyk et al. (19) revealed only abdominal pain and constipation were reported by the participants, and no significant differences between groups in these symptoms were found during follow-up as well as other adverse events. The remaining three studies (17, 21, 22) did not describe side effects.

## Discussion

Our results showed that, compared with regular diet, diet interventions can significantly promote clinician-reported core symptoms of children with ASD (*P* = 0.0008). The results of subgroup analysis indicated that: Firstly, we observed the benefits of KD dietary interventions in core symptoms of ASD (*P*_*KD*_ = 0.0003), and no significant improvement of GFD and GFCF treatment (*P*_*GFD*_ = 0.09 and *P*_*GFCF*_ = 0.06); Secondly, it suggested that the effectiveness compared between the different duration of interventions showed no significant difference (SMD_ <6M_ of −0.47, SMD_≥6*M*_ of −0.53, P_<6M_ = 0.002, P_≥6*M*_ = 0.03); Thirdly, it indicated that GFD intervention improved the symptoms of social behaviors (*P* = 0.02), however, the cognition, communication, and stereotypical behaviors symptoms showed no improvement (all *P* > 0.05); Finally, all studies reported that there were no extra safety concerns compared with the regular diet, and even had improvement in GI discomfort. These data thus lend support to dietary interventions as alternative therapies that can improve the management of ASD.

The prevalence of ASD has steadily increased over the past decades (35). The etiology of ASD remains unknown. Various risk factors have been implicated in the causation of ASD, including genetic, epigenetic, environmental risk factors (prenatally and postnatally changes) (36, 37). The effectiveness of currently available therapies for ASD is limited and uncertain, so many families search for alternative methods (38). In this situation, dietary interventions are chosen by many families, because parents believe these therapies can improve behavioral outcomes and may reduce the burden of other co-occurring conditions, such as GI issues (39). Although there is no definitive evidence on the effectiveness of dietary treatment for ASD, it has been reported that up to 33% of parents hide information on nutritional treatments/supplements from their physician (40). Furthermore, up to 50% of children with ASD have a dietary intervention that was prescribed by a medical professional (41). At present, the most commonly used dietary interventions are gluten-free and casein-free, ketogenic, and specific carbohydrate diets, as well as probiotics, polyunsaturated fatty acids, and dietary supplements (14).

The main result of the meta-analysis showed a positive effect of diet therapy for ASD symptoms [consistent with part of previous reviews (25, 42)], no correlation with the length of the interventions. Besides, the groups on GFD diets show improvement in social behaviors compared to control groups. However, another part of systematic reviews suggests that there is little evidence showing the benefits of a GFCF diet for the symptoms of ASD in children and point out the importance of identifying the best responders to the GFCF diet (16, 43, 44).

Via subgroup analysis, we observed that there was no statistical significance in the effectiveness of GFD and GFCF, which was inconsistent with the RCTs by Ghalichi et al. (18) and agreed with the result of Piwowarczyk et al. (19). This may be due to imperfect blinding and differences in measurement criteria. The benefit of the KD diet supports previous studies (20, 45, 46). Despite this, the limitation is very obvious, such as small sample sizes, subjective nature of parent observations reporting symptoms, difficulty adhering to the diet therapy, lack of uniform measurement standards, variation in dietary composition and dosage between different experiments. Most of the included studies reported no significant side effects, but we should also be vigilant for possible situations, such as gastrointestinal discomfort, weight loss, malnutrition, eating disorders during the dietary intervention (47).

In terms of the quality of the literature and study design, no attrition or reporting bias was found among the seven RCTs, and only in two studies was the randomized method described well. Only one study used double-blinding and another one was performed single-blinding, all of the remaining five studies did not describe blind methods, which can lead to some degree of bias. Most results of sensitivity analysis showed stability effect, except for the GFCF diets subgroups were influenced by one study separately.

## Strength and Limitations

The strength of the meta-analysis is that it is the first study to analyze the effectiveness of diet therapies including GFCF diet, GFD, and KD for children with ASD, quantitatively and systematically. However, the limitations are obvious too. First, the diagnostic criteria for inclusion of children with ASD and scales for symptom assessment varied between trials. Second, the dietary regulatory process of most experiments and assessment of partial functional levels were done by parents, which meant a certain subjectivity. Third, there were no uniform diet standards, hence the amount and composition of the diets used were different among the intervention groups, and the adherence to the dietary intervention was poor leading to high dropout rates. Fourth, the sample sizes of the included RCTs were relatively small. Finally, due to no contact with the authors of the included studies for further information, the results are based only on data published, and no gray literature is included.

## Conclusions

In summary, our study shows that diet therapies are beneficial for children with ASD. These interventions can significantly ameliorate core symptoms, and the GFD diets is conducive to improving social behaviors. The result partly provides evidence for dietary treatment becoming a therapeutic approach for ASD. However, the limitations of the literature calls for a more cautious interpretation of the results. Therefore, more well-designed, larger sample sizes and multicenter involvement studies are needed to validate the above conclusions.

## Data Availability Statement

The raw data supporting the conclusions of this article will be made available by the authors, without undue reservation.

## Author Contributions

YY, JH, and XC contributed to the study design, provided the methodology for the study, edited and revised the manuscript. JF, XW, LP, and CG were involved in database searches, including screening studies, extracting final details, assessment, and analysis. CC confirmed the authenticity of all the raw data and revised the manuscript. All authors read and approved the final manuscript.

## Funding

The present study was supported by the National Natural Science Foundation of China (grant number 81771589), the Public Health and Technology project of Tianjin (grant number TJWJ2021ZD007), and the Public Health and Technology project of Tianjin (grant number ZC20120). The sources of support had no influence on the content of the manuscript.

## Conflict of Interest

The authors declare that the research was conducted in the absence of any commercial or financial relationships that could be construed as a potential conflict of interest.

## Publisher's Note

All claims expressed in this article are solely those of the authors and do not necessarily represent those of their affiliated organizations, or those of the publisher, the editors and the reviewers. Any product that may be evaluated in this article, or claim that may be made by its manufacturer, is not guaranteed or endorsed by the publisher.
