# Supplementary material for: Efficacy and Safety of Diet Therapies in Children With Autism Spectrum Disorder: A Systematic Literature Review and Meta-Analysis
Source: Front Neurol. 2022 Mar 14;13:844117. doi: 10.3389/fneur.2022.844117 (PMC8963985; doi:10.3389/fneur.2022.844117)

**Figure. S1**| Funnel plot of the studies.


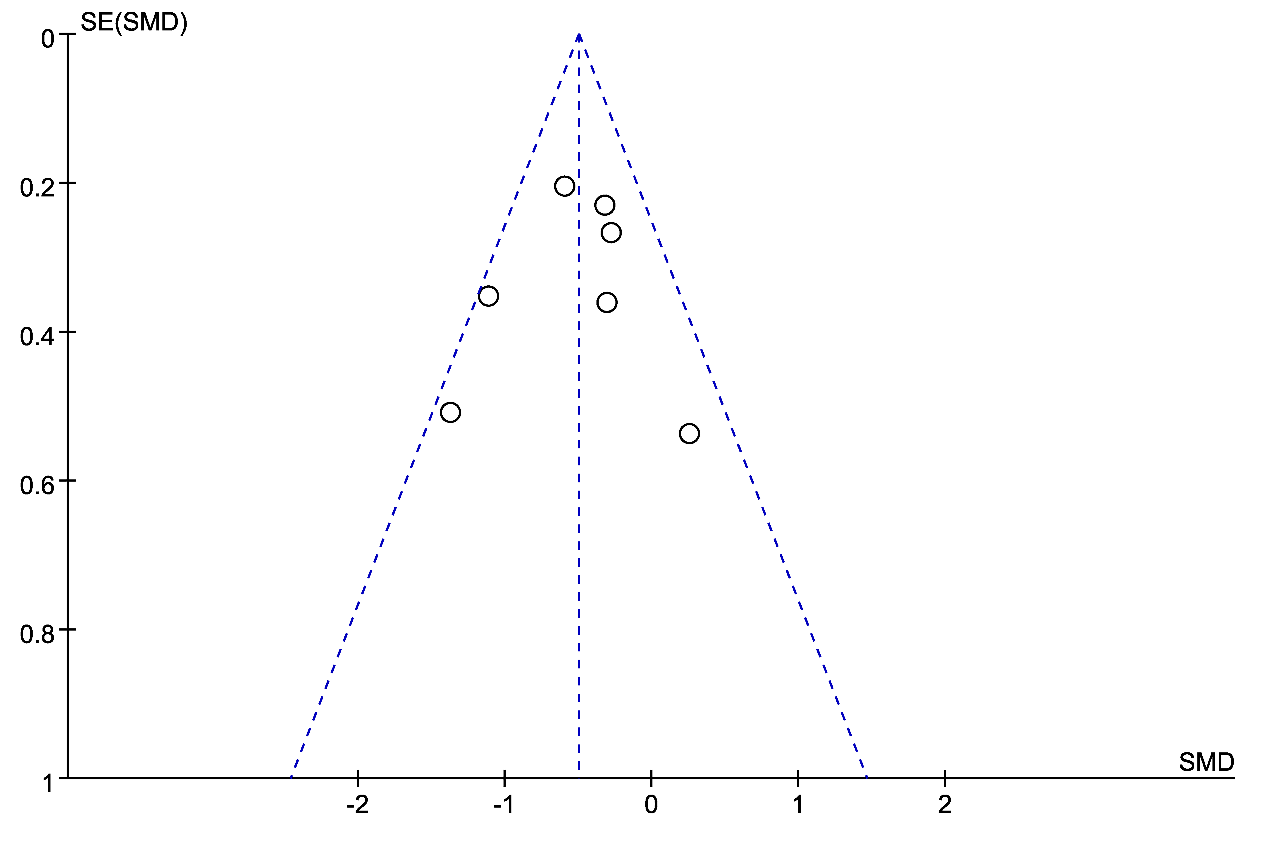


**FIGURE. S2**| Meta-analysis results and scales for cognition.

Abbreviations: ATEC, Autism Treatment Evaluation Checklist; DIPAB, a standardized Danish scheme to evaluation behavior; VABS-2, Vineland Adaptive Behavior Scale-2.


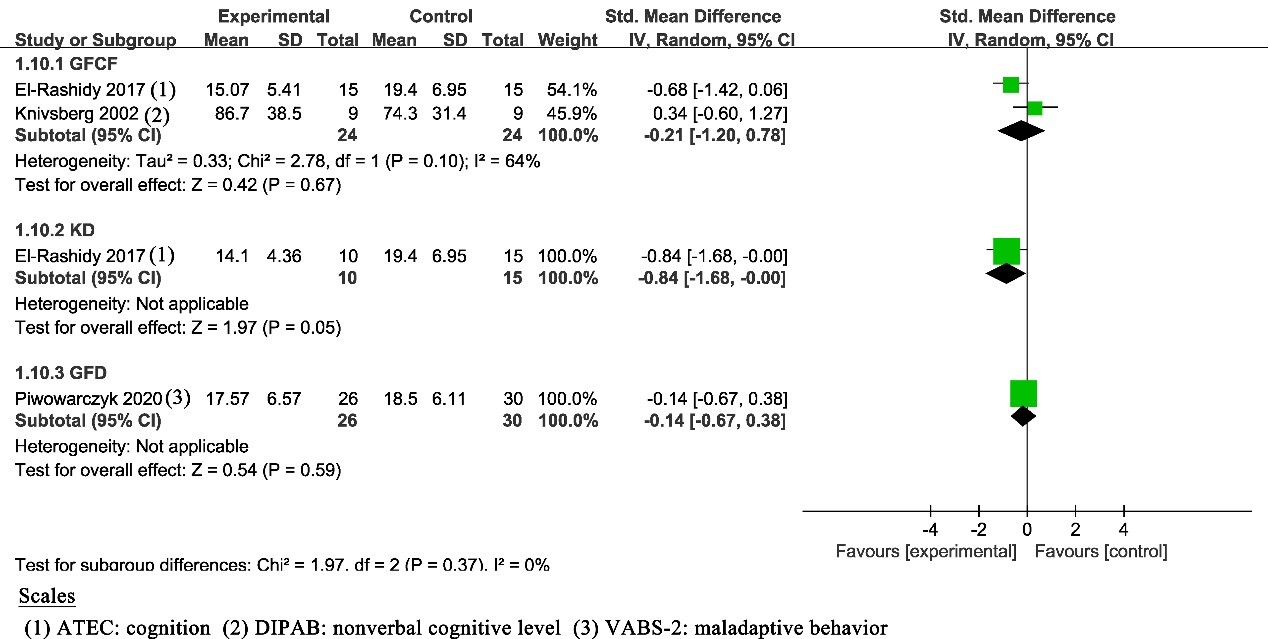


**FIGURE. S3**| Meta-analysis results and scales for communication.

Abbreviations: ECO, Ecological Communication Orientation; ATEC, Autism Treatment Evaluation Checklist; DIPAB, a standardized Danish scheme to evaluation behavior; GARS-2, Gilliam Autism Rating Scale, Second Edition; SCQ, Social Communication Questionnaire.


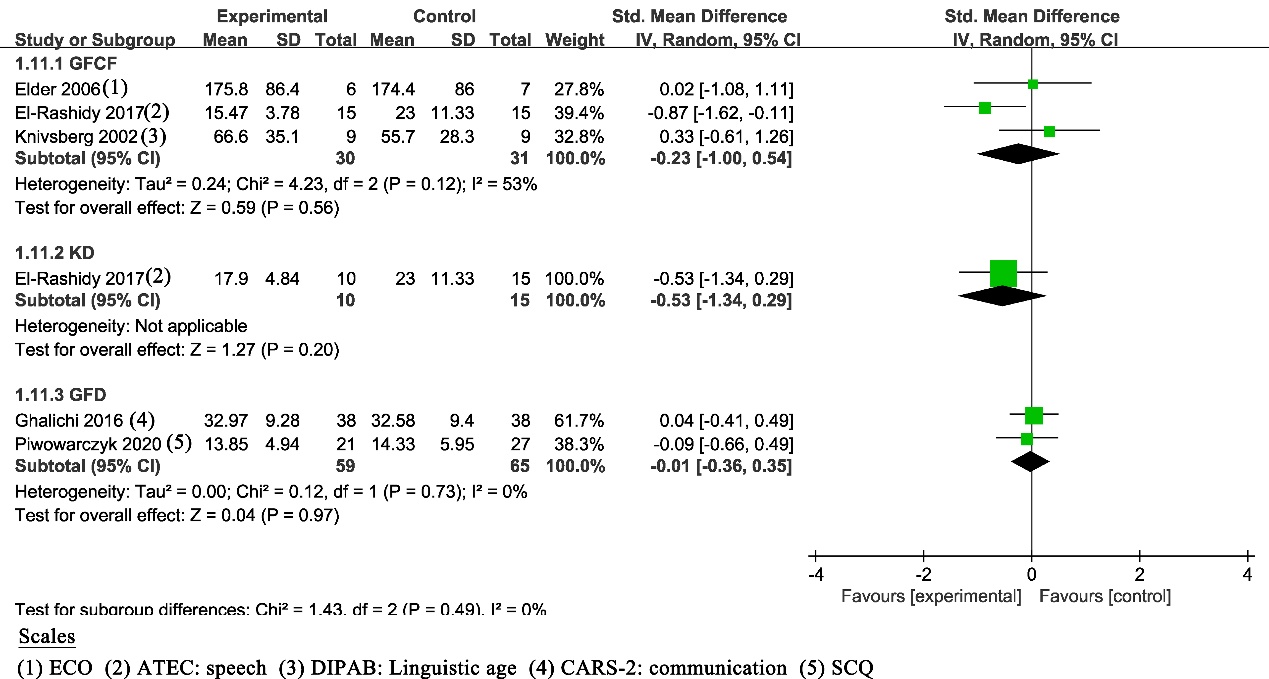


**FIGURE. S4**| Meta-analysis results and scales for stereotypical behaviors.

Abbreviations: ATEC, Autism Treatment Evaluation Checklist; DIPAB, a standardized Danish scheme to evaluation behavior; GARS-2, Gilliam Autism Rating Scale, Second Edition; ADOS-2, Autism Diagnostic Observation Schedule, Second Edition.


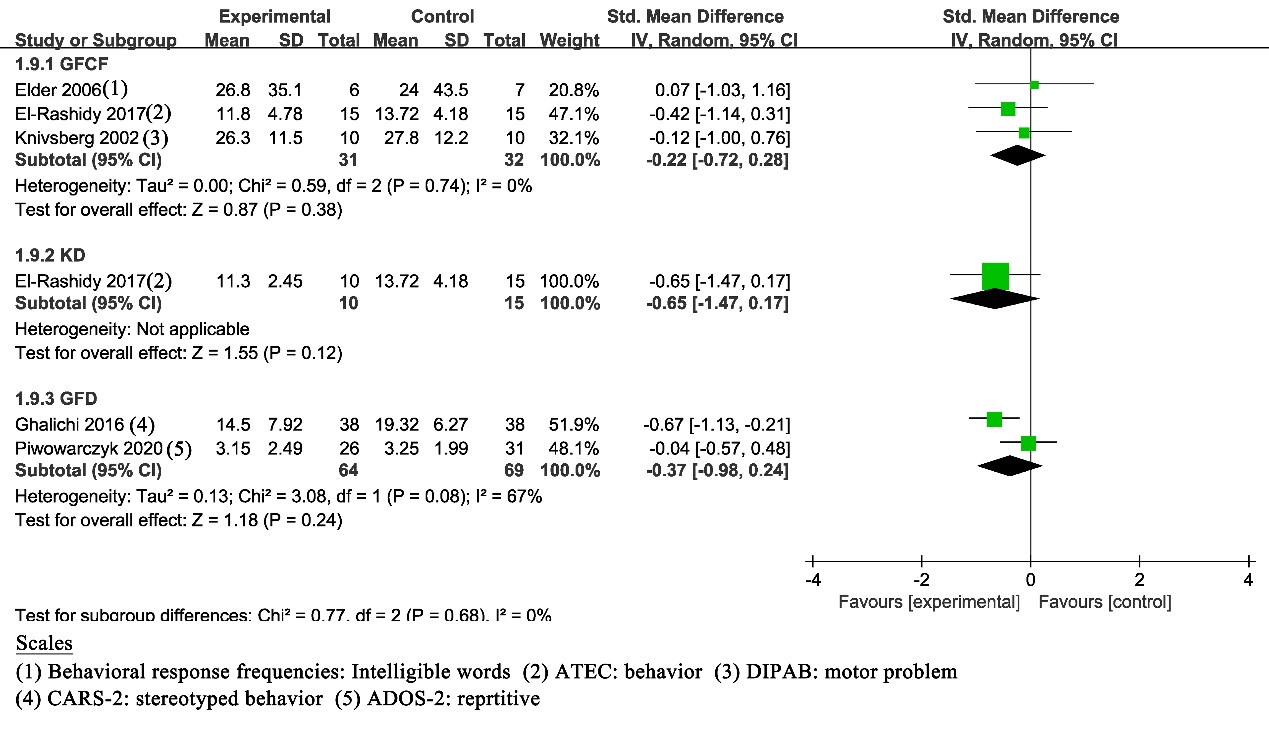

Supplement: Supplementary file 1 [file Data_Sheet_1.ZIP › Supplementary File(s)/Supplementary Figures.docx]
